# Binding and cleavage of pro-urokinase by a tegument extract of *Fasciola hepatica* newly excysted juveniles activate the host fibrinolytic system

**DOI:** 10.1186/s13567-025-01449-4

**Published:** 2025-01-25

**Authors:** Judit Serrat, María Torres-Valle, Carolina De Marco Verissimo, Mar Siles-Lucas, Javier González-Miguel

**Affiliations:** 1 Laboratory of Helminth Parasites of Zoonotic Importance (ATENEA), Institute of Natural Resources and Agrobiology of Salamanca (IRNASA-CSIC), Salamanca, Spain; 2 Molecular Parasitology Laboratory, University of Galway, Galway, Republic of Ireland

**Keywords:** *Fasciola hepatica*, newly excysted juveniles, pro-urokinase, fibrinolytic system, host‒parasite interactions

## Abstract

Plasmin, the final product of fibrinolysis, is a broad-spectrum serine protease that degrades extracellular matrix (ECM) components, a function exploited by multiple pathogens for dissemination purposes. The trematode *Fasciola hepatica* is the leading cause of fasciolosis, a major disease of livestock and an emerging zoonosis in humans. Infection success depends on the ability of *F. hepatica* newly excysted juveniles (FhNEJ) to penetrate the host intestinal wall, a process that remains incompletely understood. We have previously shown that FhNEJ are capable of binding plasminogen (PLG), the zymogen of plasmin, on their tegument surface, which leads to plasmin generation in the presence of host-derived PLG activators and subsequent degradation of laminin, a major component of the intestinal ECM. Here, we describe the interaction between a tegument extract of FhNEJ and the precursor of the urokinase-type PLG activator (pro-u-PA). We found that *F. hepatica* cathepsins B3, L3, enolase and glutathione S-transferase mediate this interaction, suggesting a multifactorial or moonlighting role for these proteins. Additionally, our results revealed that the tegument of FhNEJ contains a protease that is capable of cleaving and activating pro-u-PA into its catalytically active form, which positively impacts the capacity of the parasites to generate plasmin from the host PLG. Collectively, our findings indicate that FhNEJ interact with the host fibrinolytic system at multiple levels, reinforcing the potential of targeting this interaction as a strategy to prevent FhNEJ trans-intestinal migration and infection success.

**Supplementary Information:**

The online version contains supplementary material available at 10.1186/s13567-025-01449-4.

## Introduction

The co-option of the functions of the mammalian fibrinolytic system by infectious agents, including bacteria, fungi and parasites, is increasingly recognized as a paradigmatic example of how these organisms exploit host resources to improve their fitness and secure successful establishment within their mammalian hosts [1, 2]. The classical function of the fibrinolytic system is the degradation of fibrin clots that appear in the vascular endothelium upon injury. When these lesions have been repaired, the zymogen plasminogen (PLG) binds to lysine residues present on fibrin molecules that constitute the clot and undergoes a conformational change that exposes the target sites of its activators, the serine proteases tissue-type and urokinase-type plasminogen activators (t-PA and u-PA, respectively). Upon cleavage, PLG is converted into its catalytically active form, the serine protease plasmin, which degrades fibrin and contributes to dissolution of the clot and restoration of normal blood flow [3].

Plasmin is a broad-spectrum protease with multiple extravascular substrates, including components of the extracellular matrix (ECM), immunoglobulins and molecules of the complement system [4–6], among others. Plasmin activity is directed to the extravascular space by u-PA, which, as opposed to its blood-specific counterpart, t-PA, is expressed by a wider array of cell types, including endothelial cells, macrophages, neutrophils, intestinal epithelial cells and certain tumour cells [3, 7, 8]. u-PA, also known as urokinase, is synthesized and secreted as a precursor zymogen of 411 amino acids (pro-u-PA) that only exhibits catalytic activity after proteolytic cleavage into its active form, u-PA. Pro-u-PA cleavage and subsequent activation are mediated by different proteases, including plasmin, kallikrein, trypsin and human cathepsins B and L [9–11], and cleaved u-PA is formed by two polypeptide chains that are linked via a disulfide bond. Overall, the broad spectrum of plasmin substrates, together with the expression of u-PA by various cell types, engages the fibrinolytic system in multiple extravascular processes that require cell migration, including inflammatory responses, embryogenesis, wound healing and cancer cell dissemination [7, 9, 12]. These extravascular functions of the fibrinolytic system are exploited by parasites for migration, feeding, immune evasion and survival inside their mammalian hosts [1, 2, 13], and the helminth *Fasciola hepatica* is no exception [14–16].

*F. hepatica*, commonly known as the liver fluke, is a parasitic trematode and the most widespread causative agent of fasciolosis, a re-emerging food-borne neglected zoonotic disease that affects more than fifty million people worldwide and a large proportion of wild and domestic animals as definitive hosts [17, 18]. Globally, *F. hepatica* infection is most prevalent in cattle and sheep, with infection rates reaching up to 90%, which inflict significant economic losses on the livestock industry, estimated at up to $3.2 billion annually. These losses are primarily due to liver condemnation, severe morbidity, mortality in cases of hyper-infection, and reduced yield and quality of carcasses and animal-derived products, such as milk and wool. By doing so, *F. hepatica* infection poses major challenges to global food security, particularly in low-income countries where most pressure on increasing food production is expected to occur, provided that the population in these areas is predicted to double in the next few decades [19]. Moreover, fasciolosis also impacts agricultural practices in certain areas of the world where livestock are still employed as a source of traction power and manure in crop production [18]. Definitive hosts become infected upon ingestion of aquatic plants or water contaminated with metacercariae, which excyst and release the newly excysted juveniles (FhNEJ) upon arrival at the duodenum. FhNEJ cross the intestinal wall within the first 72 h post infection, reaching the peritoneal cavity and then the liver, where they migrate through until gaining access to the major hepatic bile ducts, where adult flukes mature and produce eggs that are shed with faeces [20, 21]. While intestinal penetration of FhNEJ is not associated with clinical symptoms, migration through the liver parenchyma of juvenile flukes and the chronic presence of adults in the biliary ducts cause allergic reactions, hepatic congestion and inflammation, bile duct hyperplasia and cholangitis, among other conditions [22, 23].

Our laboratory has recently shown that FhNEJ interact with the host fibrinolytic system by binding PLG and stimulating its conversion to plasmin, which in turn potentiates the capacity of these parasites to degrade laminin (LM), one of the major components of the intestinal ECM [16, 24]. This mechanism of co-opting host resources during the early stages of infection potentially facilitates FhNEJ penetration of the intestinal epithelium while minimizing the energy expenditure typically associated with this process. Given that u-PA is expressed by epithelial cells of the intestine, presumably to loosen intercellular junctions and facilitate epithelial cell turnover at the top of the villi [8], this study investigated the ability of FhNEJ to exploit the functions of this fibrinolytic protease for invasion and migration.

## Materials and methods

### In vitro excystment of *F. hepatica* metacercariae and protein extraction of the tegument-enriched fraction

FhNEJ were obtained by in vitro stimulation of the excystment of *F. hepatica* metacercariae (Ridgeway Research Ltd., St Briavels, UK) as previously described [16]. First, CO_2_ (99.5% purity) was bubbled in 10 mL of ice-cold distilled water for 30 s and supplemented with sodium dithionite to a final concentration of 0.02 M. This solution was added to five thousand metacercariae and incubated for one hour at 37 °C. The metacercariae were then washed twice with distilled water, resuspended in 5 mL of Hank’s balanced salt solution (Sigma, St. Louis, MO, USA) supplemented with 10% lamb bile (obtained from a local abattoir) and 30 mM HEPES (Sigma), pH 7.4, and incubated for three to 5 h at 37 °C. FhNEJ were manually recovered under a stereoscope every hour after the addition of excystment media and incubated at 37 °C and 5% CO_2_ for three hours in plates containing 4 mL of RPMI-1640 culture media (Thermo Fisher Scientific, Waltham, MA, USA) supplemented with 30 mM HEPES (Sigma), 0.1% glucose (Sigma), and 50 µg/mL gentamycin (Sigma) to induce recovery [25]. A total of 1839 FhNEJ were subjected to protein extraction of the tegument-enriched fraction (FhNEJ-Teg), which was performed in 300 µL of PBS containing 1% Nonidet P40 substitute (Sigma), as previously described [16, 26], in the absence of protease and phosphatase inhibitors. The protein concentration was 0.42 mg/mL, as determined using a Pierce BCA protein assay kit (Thermo Fisher Scientific).

### u-PA and pro-u-PA binding assays

u-PA and pro-u-PA binding to FhNEJ-Teg or recombinant proteins was assessed by enzyme-linked immunosorbent assay (ELISA) as previously described [16]. Briefly, microtiter 96-well plates (Thermo Fisher Scientific) were coated overnight at 4 °C with 0.5 µg of FhNEJ-Teg or 1 µg of recombinant *F. hepatica* cathepsin zymogens B3 (FhCB3) and L3 (FhCL3), glutathione S-transferase (FhGST) or enolase (FhENO) (a kind gift from Prof. J.P. Dalton) diluted in 200 µL of carbonate buffer (15 mM Na_2_CO_3_, 35 mM NaHCO_3_, pH 9.6). Wells coated with 1% bovine serum albumin (BSA) were used as negative controls. After three washes in PBS containing 0.05% Tween-20 (PBST), the wells were blocked with either 1% BSA or 1% milk in PBS for 30 min at 37 °C, and increasing amounts (0 µg to 1 µg) of human pro-u-PA (Abcam, Cambridge, UK) or human u-PA (Sigma) diluted in blocking solution were added to the wells and incubated for one hour at 37 °C. A competition assay was performed in parallel by adding 50 mM of the lysine analogue 6-aminocaproic acid (ε-ACA) (Sigma) to the wells during incubation with u-PA and pro-u-PA. After three washes with PBST, the wells were incubated for one hour at 37 °C with an anti-human-u-PA primary antibody raised in sheep (Innovative Research, Novi, MI, USA) diluted 1:500 in blocking solution, followed by incubation for one hour at 37 °C with a horseradish (HRP)-conjugated anti-sheep secondary antibody (Sigma) diluted 1:1000 in blocking solution. The wells were washed three times with PBST between and after antibody incubation. Finally, the binding of u-PA and pro-u-PA was verified by adding 100 µL per well of substrate buffer (25 mM citric acid, 45 mM Na_2_HPO_4_, 0.04% H_2_O_2_, pH 5.0) containing 1.5 mM of the chromogenic substrate ortho-phenylene-diamine (OPD) (Sigma). The wells were incubated at room temperature in the dark, and the reaction was stopped after 15 min by adding an equal volume of 3 N sulfuric acid. Optical density (OD) was measured at 492 nm with a Multiskan GO spectrophotometer (Thermo Fisher Scientific). The assay was performed twice in technical triplicates. The values of every condition assayed were blanked to the mean of the condition containing 0 µg of pro-u-PA to eliminate background signals from the binding of the primary antibody to the proteins used for coating. Primary antibody specificity for both pro-u-PA and u-PA was tested under the abovementioned conditions in wells coated with 250 ng of pro-u-PA (Abcam) or u-PA (Sigma). The ability of ε-ACA to block lysine-dependent interactions was tested by coating the wells with 0.5 µg of FhNEJ-Teg followed by incubation with 2 µg of human PLG in the presence or absence of 50 mM ε-ACA. PLG binding was detected following standard ELISA procedures with an anti-PLG-specific primary antibody, as described elsewhere [16].

### Bidimensional (2D) electrophoresis of FhNEJ-Teg

The FhNEJ-Teg extract (80 µg) was purified using the ReadyPrep 2-D Cleanup Kit (Bio-Rad, Hercules, CA, USA) to precipitate proteins, and protein pellets were resuspended in 250 µL of rehydration buffer [7 M urea, 2 M thiourea, and 4% 3-[(3-cholamidopropyl) dimethylammonio]-1-propanesulfonate (CHAPS)]. The extract was then divided into two aliquots of 125 µL, supplemented with 0.05 M dithiothreitol (DTT) (Sigma) and 0.2% ampholyte pH 3–10 (Bio-Rad), and added to two 7 cm ReadyStrip IPG strips (40 µg per strip) with a linear pH range of 3–10 (Bio-Rad) for passive rehydration overnight at 20 °C in Protean IEF Cell equipment (Bio-Rad). Isoelectric focusing (IEF) was performed with Protean IEF Cell equipment (Bio-Rad) at 20 °C under the following conditions: 15 min at 250 V, two hours at 4000 V and 16 000 V/h at 4000 V; a maximum amperage of 50 µA per strip was used. Next, the proteins in the strips were reduced with DTT (0.02 g/mL) and alkylated with iodoacetamide (0.0025 g/mL) for 10 min or 15 min at room temperature (both DTT and iodoacetamide were diluted in equilibration buffer containing 6 M urea, 2% SDS, 1.5 M Tris–HCl pH 8.8, 30% glycerol and bromophenol blue). The separation of the second dimension (by molecular weight) was performed in 12% acrylamide‒sodium dodecyl sulphate (SDS) gels following standard procedures. One of the gels was immediately stained with silver using in-house-prepared reagents following standard procedures (excluding formaldehyde and glutaraldehyde from the formulations to ensure compatibility with subsequent analysis by mass spectrometry) and imaged using a Chemidoc gel-imaging system (Bio-Rad). The proteins contained in the second gel were electrotransferred onto a nitrocellulose membrane for detection of pro-u-PA binding by ligand blotting (see the next section).

### Detection of pro-u-PA-binding proteins by ligand blotting

The proteins in the 2D gel were electrotransferred onto nitrocellulose membranes using a constant amperage of 400 mA for 90 min at 4 °C. The blots were blocked for 1 h at room temperature with 2% BSA diluted in PBST and incubated overnight at 4 °C with 2 µg/mL pro-u-PA (Abcam) diluted in blocking solution. After washing in PBST, pro-u-PA binding was detected by adding an anti-u-PA primary antibody raised in mice (Santa Cruz Biotechnology, Dallas, TX, USA) diluted 1:500, followed by incubation with an HRP-conjugated anti-mouse secondary antibody (Sigma) diluted 1:2000. The antibodies were diluted in blocking solution and incubated for 90 min at 37 °C. Protein spots bound to pro-u-PA were verified by enhanced chemiluminescence (Clarity Western ECL Substrate, Bio-Rad) and imaged on a ChemiDoc MP Imaging system (Bio-Rad). PDQuest Software v.8.0.1 (Bio-Rad) was used to match spots between the silver-stained gel and the corresponding blot and to estimate the experimental molecular weight (MW) and isoelectric point (*pI*) values of every spot.

### Spot analysis by liquid chromatography coupled with tandem mass spectrometry (LC‒MS/MS)

Individual protein spots in the silver-stained gels were manually excised under sterile conditions and sent for proteomic analysis at the proteomics facility of the Central Support Service for Experimental Research (SCSIE, University of Valencia), as previously described [16]. Protein identification was performed via the software ProteinPilot v5.0 (ABSCIEX, Framingham, MA, USA) to search the UniProt-Tremmatoda database (200604). Only proteins belonging to *F. hepatica* and having an unused value ≥ 2 (meaning confidence of identification ≥ 99%) were used for subsequent analyses, and protein isoforms were manually grouped to facilitate downstream data interpretation. Clades of cathepsin L entries with unspecified descriptions were assigned on the basis of similarity to canonical cathepsin L prosegment sequences via multiple sequence alignment with Clustal Omega [27], and those corresponding to cathepsin B entries were determined by phylogenetic comparison of the entire protein sequence [16]. Protein sequences were retrieved via UniProt ID mapping, and Gene Ontology (GO) analysis was subsequently performed via OmicsBox v3.0.30 software (Biobam Bioinformatics, Valencia, Spain).

### In vitro activation of *F. hepatica* cathepsin zymogens

The activation of *F. hepatica* recombinant cathepsin L1, L2, L3 and B2 was performed by mixing zymogens in an appropriate volume of citrate–phosphate (C-P) activation buffer (0.1 M citrate phosphate, 100 mM NaCl, 2 mM DTT, 10 µg/mL dextran sulphate, pH 4.5) [28] such that the final concentration of active enzyme after incubation for two hours at 37 °C was 50 µM. Zymogen activation was verified by resolving an aliquot of the activated mixture on a precast 4–20% Bis–Tris gel (GenScript, Piscataway, NJ, USA), followed by silver staining via custom-prepared reagents and standard procedures. In parallel, zymogen activation was assayed as previously described [29] in an enzymatic assay using a cathepsin-specific fluorogenic substrate (Z-Gly-Pro-Arg-AMC; Bachem, Bubendorf, Switzerland). Briefly, activated cathepsins (7.5 mM) were mixed in 100 µL of sodium acetate reaction buffer (100 mM sodium acetate, 1 mM EDTA, 1 mM DTT, 0.01% Brij-35, pH 7.0) containing 20 µM fluorogenic substrate, and their hydrolytic activity was monitored over time at 37 °C as fluorescence relative units (RFU) in a FluoStar Omega fluorimeter (BMG LabTech, Ortenberg, Germany) using excitation/emission wavelengths of 380/460 nm. The broad-spectrum protease inhibitor E-64d at 100 μM (Sigma) was used to control that the recorded fluorescent signal was specific to cathepsin proteolytic activity. All the reactions were performed in triplicate.

### Pro-u-PA activation assays

This assay was performed in 96-well plates (Thermo Fisher Scientific) in a final volume of 100 µL by measuring the amidolytic activity of generated u-PA on a u-PA-specific chromogenic substrate. In each well, 0.01 µM pro-u-PA (Abcam) was mixed with 0.5 mM pyro-Glu-Gly-Arg-pNa chromogenic substrate (5-Diagnostics, Basel, Switzerland) together with increasing amounts of FhNEJ-Teg (1–3 µg) or 0.05 µM recombinant activated cathepsin L1, L2, L3 or B2. Wells containing pro-u-PA (0.01 µM), plasmin (0.05 µM) and chromogenic substrates (0.5 mM) and wells containing u-PA (0.01 µM) and chromogenic substrates (0.5 mM) were used as controls for pro-u-PA cleavage and substrate specificity, respectively. The microplates were incubated for up to 24 h at 37 °C, and substrate cleavage was assessed by measuring the absorbance at 405 nm at different time points in a Multiskan GO spectrophotometer (Thermo Fisher Scientific). All the reactions were performed in triplicate, and the experiment was performed twice.

### Plasmin activity assay

This assay was performed in 96-well plates (Thermo Fisher Scientific) in a final volume of 100 µL of PBS by measuring the amidolytic activity of the generated plasmin on a plasmin-specific chromogenic substrate, as previously described [16]. Briefly, 1 µg of PLG (Origene, Rockville, MD, USA) was added to every well together with 0.01 µM pro-u-PA (Abcam) and 2 mM pyro-Glu-Phe-Lys-pNa chromogenic substrate (5-Diagnostics) in the presence or absence of 1 µg of FhNEJ-Teg. Wells containing different paired combinations of FhNEJ-Teg, PLG and pro-u-PA or individual compounds alone were used as controls for background signals. Wells containing 0.0051 µM u-PA (Sigma) (corresponding to the estimated amount of u-PA generated in wells containing FhNEJ-Teg + pro-u-PA at 24 h after incubation, as shown in Figure 3A) were used to control for unspecific activity of the u-PA generated in the wells toward the plasmin-specific chromogenic substrate. The microplates were incubated for up to 24 h at 37 °C, and substrate cleavage was assessed by measuring the absorbance at 405 nm at different time points in a Multiskan GO spectrophotometer (Thermo Fisher Scientific). All the reactions were performed in triplicate, and the experiment was performed twice.

### Statistical analysis

Plots were created with Prism 10 software (GraphPad Software, La Jolla, CA, USA), and statistical analyses were performed with either Prism 10 software (GraphPad Software) or the R Commander package [30]. Comparisons between more than two experimental groups were performed via analysis of variance (ANOVA) followed by Tukey post hoc analysis for pairwise comparisons. Unless otherwise stated, the differences were not significant.

## Results

### The tegument-enriched fraction of FhNEJ contains proteins that bind pro-u-PA

**Figure 1:**
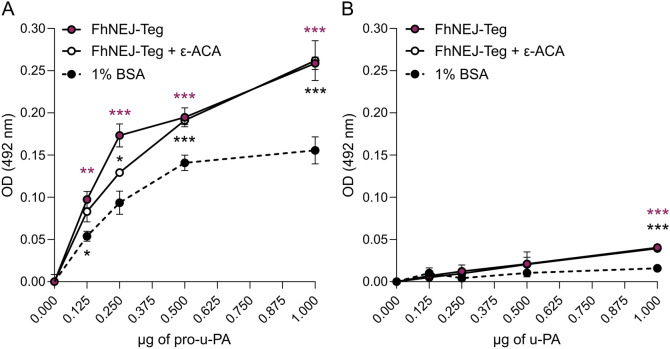
**Binding of u-PA and its precursor by FhNEJ-Teg proteins. A, B** Binding of FhNEJ-Teg proteins to pro-u-PA (**A**) or u-PA (**B**) was detected via ELISA by coating wells with FhNEJ-Teg (0.5 µg) and incubating with increasing amounts of these factors. In parallel, a condition where ε-ACA was present during pro-u-PA/u-PA incubation was included to assess whether the binding of these fibrinolytic factors to FhNEJ-Teg proteins occurs via lysine residues. Wells coated with 1% BSA served as controls for nonspecific binding to pro-u-PA/u-PA. The graphs are representative of two independent experiments. The data points indicate the means of three technical replicates ± SD, purple asterisks indicate significant differences between pro-u-PA/u-PA and the negative control (1% BSA), and black asterisks indicate significant differences between the negative control (1% BSA) and pro-u-PA/u-PA incubated in the presence of ε-ACA (**p ≤ 0.1; ***p ≤ 0.001; one-way ANOVA). Note that, in Panel B, the data points corresponding to FhNEJ-Teg and FhNEJ-Teg + ε-ACA overlap.

First, the ability of FhNEJ-Teg to bind to u-PA and its precursor, pro-u-PA, was assessed using an ELISA-based binding assay. In this experiment, wells were coated with FhNEJ-Teg and incubated with increasing amounts of either u-PA or pro-u-PA. A condition including the lysine analogue ε-ACA in the wells with pro-u-PA/u-PA was included to study whether this amino acid plays a role in the interaction of these factors with FhNEJ-Teg proteins. Our results demonstrate that FhNEJ-Teg contains proteins that bind to both the zymogen pro-u-PA and the active protease u-PA in a concentration-dependent manner, with notably lower binding affinities observed for u-PA (Figure 1). This low binding of u-PA to FhNEJ-Teg could not be explained by differential antibody affinity towards this factor, as shown in Additional file 1A. Our studies also revealed that pro-u-PA/u-PA binding occurs in a lysine-independent manner, as shown by the inability of ε-ACA to abrogate it. The ability of ε-ACA to block lysine-dependent interactions was confirmed by the incubation of FhNEJ-Teg with PLG (Additional file 1B), an interaction that is strongly inhibited by this lysine analogue [16].

### Identification of pro-u-PA-binding proteins in the tegument of FhNEJ

**Figure 2:**
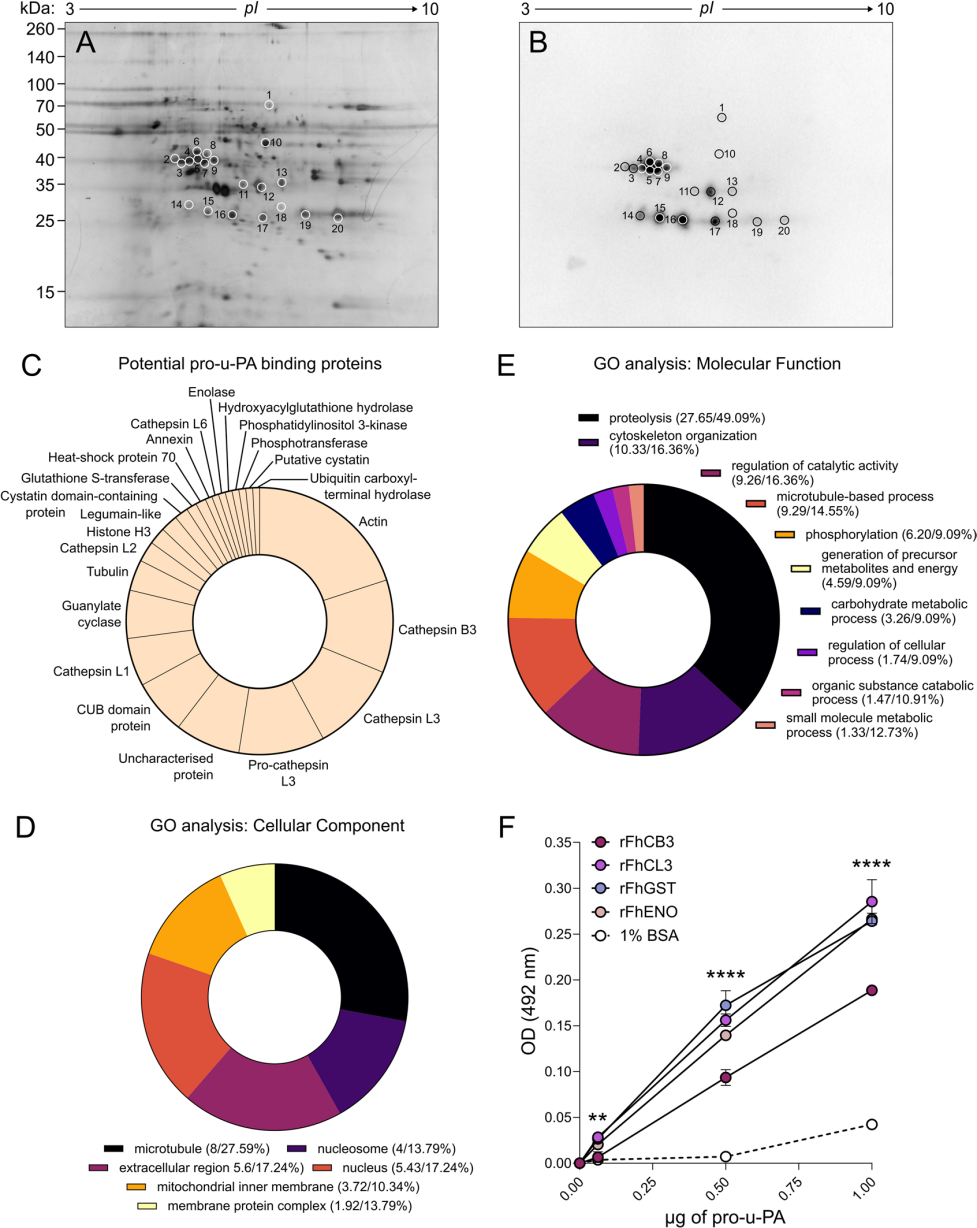
**Identification of pro-u-PA-binding proteins in FhNEJ-Teg. A, B** FhNEJ-Teg proteins were resolved by 2D electrophoresis in duplicate so that proteins in one of the gels were visualized by silver stain (**A**), and the others were transferred onto a nitrocellulose membrane to detect pro-u-PA binding by standard ligand blot procedures (**B**). Protein spots are circled and numbered. **C** Abundance distribution of the most recurrently identified proteins in the pool of potential pro-u-PA binding proteins (circled spots). **D, E** Gene ontology analysis of the potential pro-u-PA-binding proteins identified by 2D-MS in the biological process (**D**) and cellular component (**E**) categories plotted according to node score. The values in parentheses indicate the node score/percent of sequences annotated in each category. **F** Binding of the recombinant proteins FhCB3 and FhCL3, FhGST and FhENO to pro-u-PA was detected via ELISA by coating wells with the recombinant proteins (1 µg) followed by incubation with increasing amounts of pro-u-PA. The data points indicate the means of three technical replicates ± SD, and the asterisks indicate significant differences between all the recombinant proteins and the negative control (1% BSA), with identical *p *values for each comparison (**p ≤ 0.01; **** p ≤ 0.0001; one-way ANOVA), except in the wells incubated with 0.0625 µg of pro-u-PA. In this condition, the difference between FhCB3 and 1% BSA was not significant. Note that some data points overlap: those representing rFhCB3 and 1% BSA (0.0625 µg of pro-u-PA), those representing rFhCL3 and rFhGST (0.0625 µg of pro-u-PA), and those representing rFhGST and rFhENO proteins (1 µg of pro-u-PA).

To identify the pro-u-PA-binding proteins contained within FhNEJ-Teg, we combined 2D electrophoresis, ligand blotting and LC‒MS/MS. Ligand blot analysis revealed 20 protein spots within the FhNEJ-Teg extract that appeared to bind pro-u-PA (Figures 2A and B). Subsequent LC‒MS/MS analysis revealed that these spots contained 116 different protein isoforms that corresponded to 52 different proteins with the potential to bind to pro-u-PA (Additional file 2). On average, each spot contained seven proteins, with the most common proteins identified being actin; cathepsins B3 (FhCB3), L1, L2 and L3 (FhCL3); glutathione S-transferase (FhGST); heat shock protein 70; and annexin and enolase (FhENO) (Figure 2C). The sequences of 86 out of the 116 identified protein isoforms were retrieved and used for gene ontology (GO) analysis to gain insights into their cellular locations (Figure 2D) and the biological processes in which these proteins are involved (Figure 2E). This analysis revealed that putative pro-u-PA-binding proteins in FhNEJ-Teg are involved in proteolysis and cytoskeleton organization, among other processes, and are located mainly in the nucleus or associated with the extracellular space. Finally, a panel of recombinant *F. hepatica* proteins was used to validate the LC‒MS/MS results in an ELISA-based binding assay, which revealed that FhCB3, FhCL3, FhGST and FhENO bind to pro-u-PA in a concentration-dependent manner. While FhCL3, FhGST and FhENO bound to pro-u-PA with similar affinities, FhCB3 exhibited lower efficiency in pro-u-PA binding (Figure 2F). The alignments of the proteins identified by 2D-MS and the corresponding recombinant versions used for validation are provided in Additional file 3.

### FhNEJ-Teg contains proteins that activate pro-u-PA into its catalytic form

**Figure 3:**
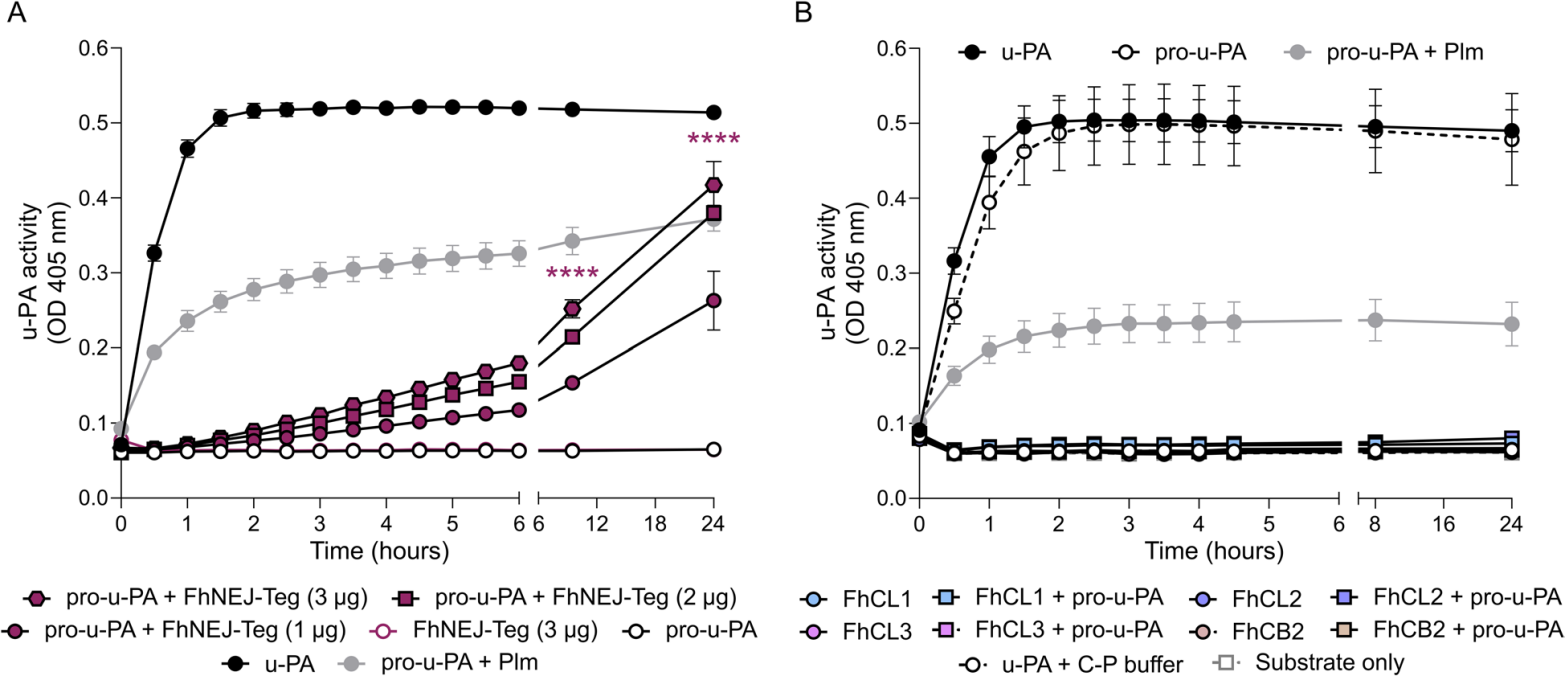
**FhNEJ-Teg stimulates the catalytic activity of pro-u-PA. A** Increasing doses of FhNEJ-Teg (1 µg to 3 µg per well) were incubated with human pro-u-PA and u-PA-specific chromogenic substrate, and u-PA generation was assessed by measuring substrate cleavage over time (absorbance at 405 nm). The figure shows a representative result of two independent experiments where the data points indicate the means of three technical replicates ± SD. Differences between all FhNEJ-Teg doses + pro-u-PA and pro-u-PA alone were significant from three hours onwards, but significance is shown only at the latest timepoints for visualization purposes (****p ≤ 0.0001; one-way ANOVA). **B** Catalytically active recombinant *F. hepatica* cathepsin peptidases L1, L2, L3 and B2 were incubated with human pro-u-PA and u-PA-specific chromogenic substrate, and u-PA generation was assessed by measuring substrate cleavage over time (absorbance at 405 nm). Wells containing u-PA alone were used to control for substrate specificity, and wells containing pro-u-PA alone were used to confirm that this zymogen lacks catalytic activity. Wells containing u-PA in the presence of C-P activation buffer were used to ensure that the components of cathepsin activation (C-P) buffer did not inhibit u-PA catalytic activity. The data points indicate the means of three technical replicates ± SDs. None of the differences between active cathepsins incubated alone or in the presence of pro-u-PA were significant (one-way ANOVA). In both panels, wells containing pro-u-PA with plasmin instead of FhNEJ-Teg serve as positive controls for u-PA generation from pro-u-PA [31].

Since pro-u-PA requires cleavage to become active, we next examined whether FhNEJ-Teg contains proteases capable of converting pro-u-PA into active u-PA. To test this hypothesis, a u-PA-specific activity assay was performed by co-incubating FhNEJ-Teg with pro-u-PA in the presence of a u-PA-specific chromogenic substrate to monitor substrate cleavage over time. Wells containing plasmin instead of FhNEJ-Teg were included as positive controls for the generation of u-PA from its precursor [31], and wells containing mature u-PA served as controls for substrate specificity. Our results revealed that, compared with the absence of the extract, FhNEJ-Teg increased u-PA activity upon incubation with pro-u-PA over time in a dose-dependent manner (Figure 3A). Importantly, such activity was not an artifact induced by FhNEJ-Teg buffer components, as verified in Additional file 4A.

We next tested the ability of recombinant *F. hepatica* cathepsins L1, L2, L3 and B2 to cleave and activate pro-u-PA. First, the activation of each cathepsin zymogen into its mature form was confirmed by SDS‒PAGE and by an enzymatic assay using a cathepsin-specific fluorogenic substrate (Additional file 4B, C) [28, 29]. The active enzymes were subsequently used to assess pro-u-PA activation in a u-PA enzymatic assay, as described above for FhNEJ-Teg. Our results revealed that none of the assayed *F. hepatica* cathepsins were capable of cleaving and activating pro-u-PA (Figure 3B) under the conditions used in this experiment. Notably, we used the same concentrations of mature cathepsins as plasmin, which serves as a positive control for u-PA generation from pro-u-PA [31], and we confirmed that u-PA activity is not inhibited by the buffer used for cathepsin activation (Figure 3B, u-PA + C-P buffer).

### Activation of pro-u-PA by FhNEJ-Teg enhances plasmin generation from the host PLG

**Figure 4:**
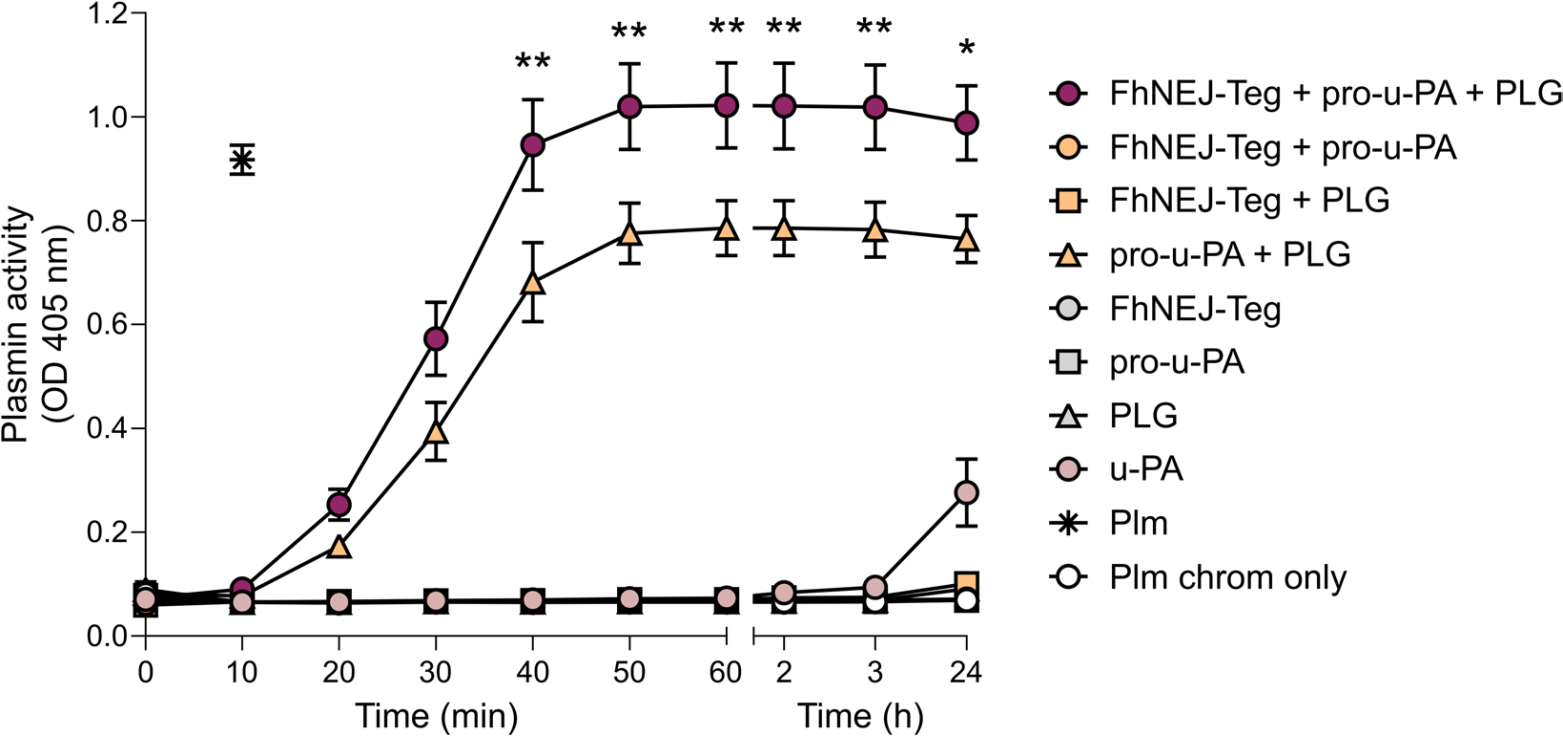
**Stimulation of u-PA generation from pro-u-PA by FhNEJ-Teg proteins enhances plasmin generation from the host PLG.** Pro-u-PA was incubated in the presence or absence of FhNEJ-Teg and PLG, and plasmin generation was assessed by measuring the cleavage of a plasmin-specific chromogenic substrate (absorbance at 405 nm) over time. Wells containing u-PA and the plasmin-specific chromogen were used to control for unspecific activity of u-PA towards this substrate, and wells containing plasmin alone were used to control for substrate specificity. The figure shows a representative result of two independent experiments, where the bars indicate the means of three technical replicates ± SD. Asterisks indicate significant differences between pro-u-PA + PLG with and without FhNEJ-Teg (***p ≤ 0.001; one-way ANOVA). Note that the data points corresponding to FhNEJ-Teg + pro-u-PA, FhNEJ-Teg + PLG, FhNEJ-Teg, pro-u-PA, PLG, u-PA and Plm chromosomes only overlap.

Finally, to test whether pro-u-PA activation by FhNEJ-Teg proteins is sufficient to trigger the fibrinolytic route in the presence of host PLG, we set up an enzymatic assay to study plasmin generation in the presence of pro-u-PA, FhNEJ-Teg and PLG. To that end, these compounds were incubated either alone or in different combinations in the presence of a plasmin-specific chromogenic substrate, and substrate cleavage over time was measured as a surrogate for plasmin generation in the wells. Although the condition containing pro-u-PA and PLG resulted in a progressive increase in substrate cleavage due to residual plasmin in the PLG stock used (Additional file 5A), the results demonstrated that the u-PA generated from pro-u-PA by FhNEJ-Teg can cleave host PLG, leading to a significant increase in plasmin generation in the wells (Figure 4) compared with those incubated without the extract. Notably, increased plasmin generation in wells containing FhNEJ-Teg, pro-u-PA and PLG was not caused by FhNEJ-Teg buffer components (Additional file 5B).

## Discussion

*F. hepatica* has evolved sophisticated mechanisms to migrate through the organism of its mammalian host, including the expression of an armoury of papain-like cysteine peptidases, known as cathepsins, that degrade tissue components and facilitate parasite invasion and establishment [32–35]. In addition to these endogenous mechanisms, our laboratory has recently shown that FhNEJ are capable of stimulating plasmin generation from host-derived PLG, which further enhances the ability of these parasites to degrade intestinal ECM components in vitro, potentially facilitating parasite migration through the intestinal wall [16, 24]. Since FhNEJ do not produce proteases capable of directly cleaving and activating PLG but rely on host-derived PLG activators to convert this zymogen into plasmin [16], we hypothesized that they might also interact with and modulate the activity of the primary intestinal PLG activator, u-PA, to further potentiate plasmin generation from circulating PLG.

To test this hypothesis, we first sought to determine whether FhNEJ interact with u-PA and its precursor, pro-u-PA. To this end, we co-incubated a tegument-enriched fraction of FhNEJ (FhNEJ-Teg) with increasing amounts of either pro-u-PA or u-PA and analysed their interaction by ELISA. We focused on FhNEJ-Teg proteins because the tegument represents the interface that is in direct contact with host tissues, and we have previously shown that this extract contains proteins that bind to host PLG and stimulate plasmin generation in the presence of the host-derived activators t-PA and/or u-PA [16, 36]. Our results showed that FhNEJ-Teg proteins interact with the precursor of u-PA in a concentration-dependent manner, an interaction that is more efficient than their binding to the active enzyme, u-PA. The observation that the binding of u-PA to FhNEJ-Teg proteins is significant only at high concentrations (1 µg) suggests that this interaction may not occur under physiological conditions. Since protein interactions between FhNEJ-Teg and PLG are mediated by kringle domains present in the PLG molecule [16], which bind to lysine residues of partner proteins, we tested whether the kringle domain of pro-u-PA also mediates the interaction with this extract. Our results showed that the interaction between pro-u-PA and FhNEJ-Teg proteins is not mediated by the kringle domain, as shown by the inability of the lysine analogue ε-ACA to abrogate it, which is not surprising considering that a kringle-deficient mutant of u-PA still binds to its physiologic receptor, the urokinase-type plasminogen activator receptor (uPAR), although with lower binding stability [37].

To determine the identity of the FhNEJ-Teg proteins that bind to pro-u-PA, we performed a 2D-MS/MS analysis of this extract, which revealed that many of them coincided with those identified as PLG-binding proteins in the past [16], such as actin, FhCL3, FhENO, FhGST and heat shock protein 70. Since 2D-MS is performed under denaturing conditions, which might expose pro-u-PA binding sites that are not available for pro-u-PA binding under physiologic conditions, we partly validated the 2D-MS/MS results in an ELISA and used recombinant versions of some of the identified proteins, including FhCB3 and FhCL3 zymogens, FhGST and FhENO. These assays are conducted under native or physiological conditions, providing a more accurate reflection of the in vivo situation. We selected these proteins on the basis of our previous observations of their ability to bind either PLG, LM, or both [16, 24, 38].

This finding indicates that the same protein is capable of establishing interactions with different components of the host fibrinolytic system. Interestingly, the ability to establish interactions with multiple fibrinolytic factors is common to other helminth proteins that interact with the fibrinolytic route [13]. This is also the case for the physiologic receptor for u-PA, uPAR, which interacts with u-PA via a central cavity that leaves the external receptor surface accessible for interaction with other proteins, such as vitronectin and integrins, thereby functioning as a hub that colocalizes all the protein effectors that need to be engaged to execute the different biological functions that are mediated by u-PA/uPAR [39]. Notably, the pro-u-PA-binding proteins identified in the present study not only coincide with those that we recently identified as PLG-binding proteins but are also capable of binding LM in vitro, one of the major components of the intestinal ECM [16, 24, 40]. By doing so, these pro-u-PA/PLG/LM-binding proteins expressed at the FhNEJ surface function as multiprotein receptors that orchestrate the fine interplay between fibrinolytic effectors and ECM proteins that are needed to focalize fibrinolysis towards tissue components of the intestinal wall, which would otherwise block FhNEJ migration.

Notably, our gene ontology analyses revealed that some of the identified pro-u-PA binding proteins in FhNEJ-Teg, such as FhENO and FhGST, are intracellular enzymes with canonical cytosolic functions. The detection of intracellular and nuclear proteins in our 2D-MS analysis could be attributed to detergent-dependent exposure of these proteins during the extraction of FhNEJ-Teg, which could accidentally enrich this extract with proteins that are not naturally present on the parasite's surface. However, previous studies have demonstrated that intracellular and nuclear proteins can be relocated to the cell surface to perform noncanonical functions, including PLG binding [41–48]. The relocation of intracellular proteins for PLG binding purposes has also been described in other parasitic helminths [13]. On the basis of this knowledge, we propose that our findings reflect a moonlighting role for *F. hepatica* enolase, GST and histones 3 and 2B rather than nonspecific protein binding or detergent-mediated accidental extraction from the tegument fraction. Protein moonlighting has supposedly evolved as a strategy to amplify the number of functions that can be executed with a stable set of proteins by simply shuffling them from one cellular location to another, which allows for their interaction with additional partner proteins (e.g., extracellular proteins) that were not available in their original location [49]. From an evolutionary perspective, the moonlighting of housekeeping and cytoskeletal proteins at the parasite surface is advantageous because these proteins are highly conserved across species and cannot be targeted by the host immune system without undesired self-recognition.

To the best of our knowledge, the mechanisms by which intracellular moonlighting proteins are translocated to the cell surface remain largely unknown. It has been proposed that this process may involve lipid binding prior to translocation to the outer membrane, interaction with partner proteins that contain signal sequences for extracellular export, or secretion as part of the cargo of extracellular vesicles. Regardless of the secretion pathway, extracellular translocation is believed to require posttranslational modifications (PTMs) and is followed by the noncovalent association of the protein with the extracellular environment via an as yet unidentified mechanism [32, 50]. Interestingly, some of the potential pro-u-PA proteins identified in this study, such as heat-shock protein 70 (Accession B1NI98) and the CUB domain protein (Accession A0A2H1C1S8), are present in FhNEJ EVs [51]. In addition, one of the FhCL3 protein isoforms (Accession Q9GRW4) identified in this work as a potential pro-u-PA binding protein appears in spots (14, 16, 17 and 20) with significantly different isoelectric points, suggesting that this protein may acquire distinct PTMs prior to its translocation to the parasite surface.

The glycolytic enzyme enolase is a paradigmatic example of a moonlighting protein that contributes to the virulence potential of very different pathogenic organisms, including parasites [13], fungi [52], bacteria [53] and even viruses [54]. Recently, *F. hepatica* enolase has been described as a moonlighting protein that is capable of interacting with PLG and the intestinal ECM protein LM [38], and we now show that this glycolytic enzyme is also capable of interacting with pro-u-PA, which together with PLG, represents a leading driver of extravascular plasmin-mediated proteolysis. In addition to FhENO, FhGST, which was identified and validated as a pro-u-PA binding protein in the present study, was also identified as a LM-binding protein in previous studies [24]. In light of these findings, the acquisition of novel protein functions that avoid de novo transcription and translation (i.e., moonlighting) represents an efficient energy-saving strategy employed by FhNEJ to rapidly adapt to the constantly changing host microenvironment and rather expend their vital energy stores in growth and metabolic reprogramming, two processes that are crucial for infection success at the earliest phases of infection [55].

Next, we wanted to address whether FhNEJ-Teg contains proteases that are capable of directly cleaving pro-u-PA and converting it to its catalytically active form, u-PA. To this end, we co-incubated FhNEJ-Teg in the presence of pro-u-PA and a u-PA-specific chromogenic substrate and monitored substrate cleavage over time as a surrogate for u-PA generation. This experiment revealed that FhNEJ-Teg potentiates u-PA-specific substrate cleavage in a dose-dependent manner, which is presumably mediated by a protease that directly cleaves pro-u-PA and activates its catalytic activity. However, the binding of pro-u-PA to its physiologic receptor, uPAR, enhances the catalytic activity of this zymogen even in the absence of proteolytic cleavage [56]. Therefore, whether the increased pro-u-PA activation that we detected in the presence of FhNEJ-Teg proteins is due to pro-u-PA cleavage and subsequent u-PA generation, the binding-mediated enhancement of pro-u-PA enzymatic activity in the absence of cleavage, or both, remains to be determined. Nonetheless, the fact that FhCL3 was identified and validated as a pro-u-PA binding protein but failed to stimulate its catalytic activity points towards the former. While the cleavage and activation of pro-u-PA for dissemination purposes is well documented in bacteria [57, 58], to our knowledge, this is the first instance of such a function being described in a helminth parasite, with only one previous study reporting t-PA activation by the blood fluke *Schistosoma mansoni* [13, 59].

Since cathepsin peptidases are highly expressed at the FhNEJ stage and some of their human orthologues can cleave and activate pro-u-PA [10, 11, 32], we tested whether the increased u-PA activity observed in our enzymatic assay was mediated by *F. hepatica* cathepsins. Owing to the technical challenges encountered during the activation of FhCB3, the ability of this protease to cleave and activate pro-u-PA could not be tested. Our studies revealed that recombinant *F. hepatica* cathepsins L1, L2, L3 and B2, in striking contrast to their human orthologues, are not involved in pro-u-PA cleavage and activation. Given that FhCLs and FhCB2 do not activate pro-u-PA and that proteases that typically cleave and activate pro-u-PA under physiological conditions are predominantly serine proteases, including plasmin, trypsin, kallikrein, and tryptase [9], it is tempting to speculate that pro-u-PA activation is carried out by a serine protease expressed in the tegument of FhNEJ. Unlike their cysteine-type counterparts, serine proteases of *F. hepatica* are poorly described, and a dipeptidyl peptidase (DPP) that shares similarity with human DPP-2 and DPP-4 is one of the very few, if not the only, serine proteases characterized in this parasite [60, 61]. Considering that human DPP-4 is a PLG receptor on the surface of certain cell types [62, 63] and that this protease was identified in previous studies by us as a potential PLG-binding protein in the tegument of FhNEJ [16], one could speculate that *F. hepatica* DPP might be the protease responsible for the pro-u-PA cleavage identified in the present study.

Our enzymatic assay revealed a significant but discrete and dose-dependent increase in pro-u-PA cleavage in the presence of FhNEJ-Teg, suggesting that the protease(s) responsible for this increase in the FhNEJ-Teg extract, if any, might be present at very low concentrations or that this function is executed by a limited number of proteases. In this scenario, minimal u-PA activity at the parasite surface might still be sufficient to generate small but localized amounts of plasmin from tegument-bound PLG [16], which, in turn, would cleave and activate surface-bound pro-u-PA [31] in a positive feedback loop that ultimately leads to potent plasmin generation in the peri-parasitic space. To test this hypothesis, we co-incubated pro-u-PA, PLG and a plasmin-specific chromogenic substrate in the presence or absence of FhNEJ-Teg to assess whether the generation of u-PA from pro-u-PA by FhNEJ-Teg proteases would stimulate PLG activation into plasmin. Although incubation of PLG with pro-u-PA resulted in some background plasmin generation, this effect was further enhanced in the presence of FhNEJ-Teg proteins, confirming that pro-u-PA cleavage and activation by FhNEJ-Teg proteases positively influence the ability of these parasites to generate plasmin from tegument-bound host PLG.

The large number of potential pro-u-PA binding proteins identified in this work indicates that FhNEJ proteins interact with pro-u-PA via highly redundant mechanisms, as is the case for their interaction with the host PLG [16]. Biochemical redundancy is a hallmark of essential biological functions, as it endows an organism with backup mechanisms to ensure the maintenance of that function even in the absence of a specific effector [64]. Although biochemical redundancy emphasizes the idea that the interaction between FhNEJ and the host fibrinolytic system might be a key process for parasite survival, it also implies that targeting this parasite‒host relationship for therapeutic or control purposes is very challenging. However, the fact that the interaction between FhNEJ and pro-u-PA does not seem to be mediated by lysine residues suggests that the interaction mechanism in this case might be more specific than that between FhNEJ-Teg and PLG and thus more easily targetable from a pharmacological or immunological perspective. In relation to this, targeting the u-PA/uPAR interaction using synthetic peptide antagonists and monoclonal antibodies has recently arisen in the oncology field as a valuable strategy to block cancer cell migration and tumour progression [65, 66]. It is tempting to speculate that similar strategies could be applied to infections by *F. hepatica* and potentially other parasites that interact with the host fibrinolytic system for migration and invasion inside their mammalian host. Theoretically, such an approach would not elicit undesirable side effects on host fibrinolysis since pronounced species specificity exists in the interaction between u-PA and its physiologic receptors [65, 67].

In conclusion, this study elucidates the interaction between FhNEJ and host-derived pro-u-PA and reveals how FhNEJ-Teg proteins enhance pro-u-PA activation through direct cleavage by an as yet unidentified protease, which, in turn, significantly increases the ability of FhNEJ to activate the fibrinolytic system on their surface. Taken together, the present results complement previous work from our laboratory that revealed that FhNEJ interact with host PLG to stimulate plasmin generation and potentiate the endogenous capacity of these parasites to degrade LM, a major component of the intestinal ECM [16, 24]. Taken together, these data reveal multilayered activation of the host fibrinolytic system on the surface of FhNEJ, which might be worth exploring for immunological or therapeutic interventions against early-stage fasciolosis.

## Supplementary Information

**Additional file 1: Related to Figure **1.** A** The specificity of the primary antibody used to detect pro-u-PA and u-PA binding was tested by ELISA. The wells were coated with either u-PA or pro-u-PA, followed by incubation with the u-PA-specific primary antibody used in the assay presented in Figure 1. **B** Detection of plasminogen (PLG) binding to wells coated with FhNEJ-Teg or 1% BSA in the presence or absence of ε-ACA (50 mM) was used to test the ability of this compound to inhibit lysine-dependent interactions [16]. In both panels, the bars indicate the means of three technical replicates ± SD, and the asterisks indicate significant differences between the indicated groups (***p ≤ 0.001, **** p ≤ 0.0001, ns, not significant; one-way ANOVA).

**Additional file 2: Related to Figure **1.** A, B** Potential pro-u-PA-binding proteins in the tegument-enriched fraction of FhNEJ identified by 2D-LC‒MS/MS. Only entries with Unused ≤ 2 and corresponding to *F. hepatica* are shown. Experimental molecular weight (MW, in kilodaltons) and isoelectric point (*pI*) values were estimated using the PDQuest Software v.8.0.1 (Bio-Rad).

**Additional file 3: Related to Figure **2**F**. Alignments of protein sequences of the recombinant proteins used for validation experiments are shown in Figure 2F, as are those identified by 2D-MS analysis (Additional file 2). The table shows percent identity matrices created by Clustal Omega [27].

**Additional file 4: Related to Figure **3. ** A** Pro-u-PA was incubated with increasing volumes of FhNEJ-Teg buffer (equivalent to those used in Figure 3A) and a u-PA-specific chromogenic substrate to control that the observed pro-u-PA activation in Figure 3A was not caused by FhNEJ-Teg buffer components. Note that the data points corresponding to pro-u-PA, pro-u-PA + FhNEJ-Teg buffer (1), pro-u-PA + FhNEJ-Teg buffer (2), pro-u-PA + FhNEJ-Teg buffer (3) and the substrate only overlap. **B, C** Activation of *F. hepatica* cathepsin zymogens was checked via an enzymatic assay involving an AMC-Gly-Pro-Arg fluorogenic substrate (B) and SDS‒PAGE followed by silver staining (C). In C, activated enzymes (arrowheads) in the absence of E-64d (−) were loaded next to their zymogen counterparts, which were incubated with C-P buffer in the presence of E64-d (+) to prevent autocatalytic activation. SDS‒PAGE gels (4–20%) were stained with silver. MW: molecular weight in kilodaltons. Notably, in both panels, the data points corresponding to the substrate only, FhCL1 + E-64d, FhCL2 + E-64d, FhCL3 + E-64d and FhCB2 + E-64d overlap.

**Additional file 5: Related to Figure **4. The residual plasmin activity in the PLG stock used in the assay shown in Figure 4 was tested by mixing PLG with a plasmin-specific chromogenic substrate. Wells containing plasmin instead of PLG served as positive controls for substrate cleavage. The bars indicate the means ± SD of three technical replicates, and the asterisks indicate significant differences (*p ≤ 0.05; **** p ≤ 0.0001; one-way ANOVA). **B** Pro-u-PA + PLG were incubated with an equivalent volume of FhNEJ-Teg buffer as that used for FhNEJ-Teg in Figure 4 and a plasmin-specific chromogenic substrate to confirm that the registered plasmin generation in Figure 4 was not caused by FhNEJ-Teg buffer components.

## Data Availability

The datasets generated during and/or analysed during the current study are available in the Zenodo repository, https://zenodo.org/records/13359577.
