# Supplementary figures and images for: Binding and cleavage of pro-urokinase by a tegument extract of Fasciola hepatica newly excysted juveniles activate the host fibrinolytic system
Source: Vet Res. 2025 Jan 25;56:20. doi: 10.1186/s13567-025-01449-4 (PMC11762853; doi:10.1186/s13567-025-01449-4)

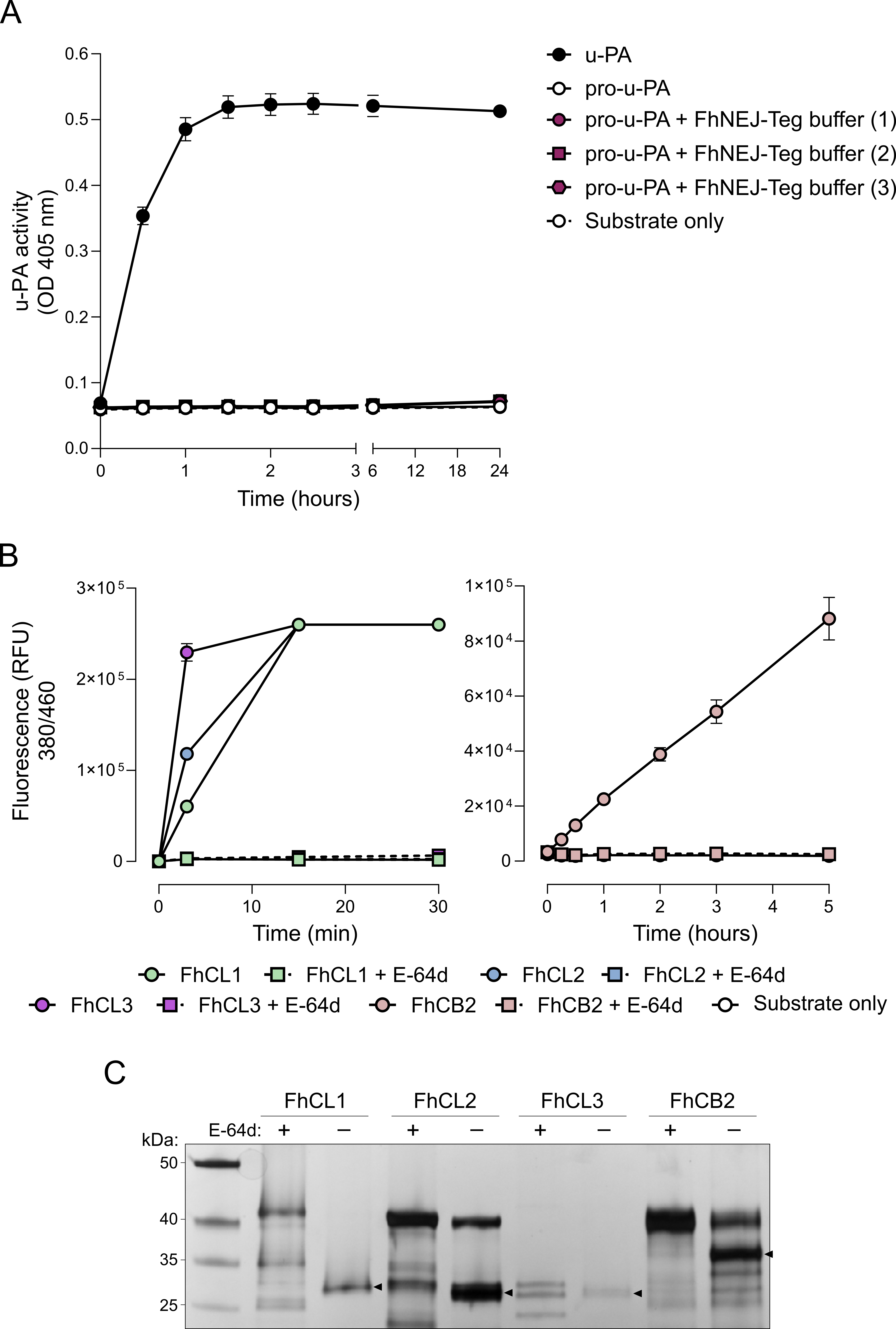

Supplement: Supplementary file 4 — Additional file 4: Related to Figure 3. A Pro-u-PA was incubated with increasing volumes of FhNEJ-Teg buffer (equivalent to those used in Figure 3A) and a u-PA-specific chromogenic substrate to control that the observed pro-u-PA activation in Figure 3A was not caused by FhNEJ-Teg buffer components. Note that the data points corresponding to pro-u-PA, pro-u-PA + FhNEJ-Teg buffer (1), pro-u-PA + FhNEJ-Teg buffer (2), pro-u-PA + FhNEJ-Teg buffer (3) and the substrate only overlap. B, C Activation of F. hepatica cathepsin zymogens was checked via an enzymatic assay involving an AMC-Gly-Pro-Arg fluorogenic substrate (B) and SDS‒PAGE followed by silver staining (C). In C, activated enzymes (arrowheads) in the absence of E-64d (−) were loaded next to their zymogen counterparts, which were incubated with C-P buffer in the presence of E64-d (+) to prevent autocatalytic activation. SDS‒PAGE gels (4–20%) were stained with silver. MW: molecular weight in kilodaltons. Notably, in both panels, the data points corresponding to the substrate only, FhCL1 + E-64d, FhCL2 + E-64d, FhCL3 + E-64d and FhCB2 + E-64d overlap. [file 13567_2025_1449_MOESM4_ESM.png]
